# Vascular Phenotyping with Photoacoustic and Ultrasound Imaging in Cardiovascular Disease: Recent Innovations

**DOI:** 10.1007/s11936-026-01162-1

**Published:** 2026-10-12

**Authors:** Muhang He, Xiaoxi Zhuang, Rui Cao

**Affiliations:** 1 Department of Biomedical Engineering, Case School of Engineering, School of Medicine, Case Western Reserve University, Cleveland, OH USA; 2 Case Comprehensive Cancer Center, Case Western Reserve University, Cleveland, OH USA

**Keywords:** Photoacoustic imaging, Ultrasound imaging, Cardiovascular disease, Vascular phenotyping

## Abstract

**Purpose of Review:**

Photoacoustic and ultrasound imaging together resolve vascular structure, wall biomechanics, blood flow, oxygenation, and tissue composition without ionizing radiation or iodinated contrast. This review summarizes what each modality can now measure in patients and asks which of these measurements are close enough to clinical use to inform cardiovascular management decisions.

**Recent Findings:**

Recent advances have expanded photoacoustic imaging toward faster, higher-resolution functional vascular phenotyping, including clinically relevant biomarkers and co-registered ultrasound imaging. Ultrafast and super-resolution ultrasound now quantify vascular biomechanics, hemodynamics, and microvasculature, with ultrasound localization microscopy entering clinical studies. Wearable ultrasound has also been validated for blood-pressure monitoring across diverse care settings, alongside increasingly quantitative feature extraction.

**Summary:**

Ultrasound and photoacoustic imaging provide complementary vascular information: ultrasound offers depth, speed, and continuous access, while photoacoustic imaging adds compositional and oxygenation contrast. Despite limitations in quantitative accuracy, imaging depth, and system standardization, emerging patient studies now span super-resolution vascular mapping, functional biomarkers, and wearable monitoring. Their integration offers a potential route toward continuous, operator-independent vascular phenotyping for clinical decision-making.

## Opinion Statement

Neither photoacoustic nor advanced ultrasound imaging is ready to replace a decision that is currently made with conventional ultrasound or computed tomography angiography. The more useful question is which specific decisions these modalities could answer that existing imaging does not address adequately. In our view, one of the strongest near-term cases is functional assessment in cardiovascular disease.

Ultrasound localization microscopy is an advanced ultrasound development, having mapped myocardial and deep cerebral microvasculature in patients, but its regulatory position is unresolved: approval of a microbubble agent for an established indication does not extend to a new imaging application, and evidence specific to that use will be required. Wearable ultrasound systems are best understood as monitoring rather than diagnostic tools, and the wearable blood pressure work validated across home, outpatient, catheterization-laboratory, and intensive-care settings is the clearest demonstration that continuous vascular measurement can be implemented across real-world care settings. Photoacoustic characterization of plaque composition remains at pilot scale, and molecular imaging of plaque inflammation remains preclinical; both are promising, but neither should be described as clinically actionable.

What the field most needs is not further gains in resolution but linkage to endpoints that already inform clinical decision-making. We would encourage investigators to design the next generation of studies around a specific decision, such as revascularization versus supervised exercise therapy in claudication, or intensification of lipid-lowering therapy in an asymptomatic carotid plaque, and to power them accordingly. Combining the two modalities is technically straightforward and physiologically sensible, but it will matter clinically only if the resulting phenotype changes what is done for a patient.

## Introduction

As one of the leading causes of death, cardiovascular disease (CVD) accounted for approximately 19.8 million deaths in 2022 [1]. The clinical difficulty is less in recognizing advanced disease than in measuring the vascular changes that precede it. Onset and progression of CVD are often accompanied by vascular alterations in vascular morphology and mechanical properties, hemodynamics, blood oxygenation, and vessel-wall composition, and these quantifiable characteristics constitute vascular phenotypes. They may not appear simultaneously. For example, impaired endothelium-dependent vasodilation may precede detectable morphological changes in the vessel wall during early atherosclerosis [2], whereas luminal narrowing, flow limitation, and a lipid-rich necrotic core emerge only as the disease advances [3]. Which phenotype can be measured, and how early, therefore determines which decisions imaging can support, from primary prevention and risk stratification through the assessment of treatment response and rehabilitation monitoring.

No single modality captures all these phenotypes, because each is built around one contrast mechanism. Vessel-wall motion and intraluminal flow demand high spatial and temporal resolution, while the assessment of blood oxygenation and vascular composition demands contrasts sensitive to specific tissue constituents. Clinical practice therefore combines modalities, each with a limitation that matters for serial assessment. Computed tomography angiography (CTA) delineates the vascular lumen, stenosis, and calcification quickly, but ionizing radiation and iodinated contrast agents limit repeat examinations [4]. Digital subtraction angiography (DSA) provides high spatial and temporal resolution and allows concurrent endovascular intervention, but its invasiveness rules out screening and serial monitoring [5]. Magnetic resonance imaging (MRI) avoids ionizing radiation and can assess the vessel wall, blood flow, and selected tissue components [6, 7]. Nevertheless, acquisition time, cost, and accessibility constrain its use for rapid or repeated imaging. Positron emission tomography (PET) detects inflammation, metabolism, and specific molecular activities with high sensitivity, at coarse spatial resolution and with radioactive tracers [8]. None of them combines the three properties that repeated vascular phenotyping requires: freedom from ionizing radiation, acquisition fast enough for dynamic and bedside use, and sensitivity to tissue composition.

Ultrasound and photoacoustic (PA) imaging are the two non-ionizing modalities that have most extended noninvasive vascular phenotyping, and they rely on different physical mechanisms. Ultrasound resolves vascular structure, the vessel wall, and intraluminal flow at high temporal resolution [9–11]. Conventional ultrasound is insensitive to slow microvascular flow, but ultrafast and super-resolution methods have made vascular mechanics, complex flow, and the microcirculation quantitatively accessible [9, 10, 12, 13]. PA imaging, also termed optoacoustic imaging sometimes, exploits differences in the optical absorption of hemoglobin, lipids, and other chromophores to report blood oxygenation and vascular composition [14, 15]. Because the two share a detection mechanism via ultrasound transducers, PA integrates readily with ultrasound to give spatially co-registered anatomical and functional information, although it is also used on its own [16]. Imaging depth, quantitative accuracy, and system standardization still bound how far PA reaches, and we return to them in the closing sections.

In this review, we use CVD in its broad sense, covering disorders of the heart and blood vessels. We include techniques demonstrated outside the cardiovascular setting if they address shared vascular biology or can be directly translated to cardiovascular applications. The sections that follow take five phenotypes in turn: vascular architecture, wall mechanics, hemodynamics, oxygenation, and plaque composition. We then turn to the two developments that determine whether these measurements can be made repeatably in practice, wearable systems and quantitative feature extraction.

## Treatment Options

### Imaging the Vascular Phenotype

#### Vascular Architecture: From the Single Capillary to the Whole Limb

Vessel geometry is the basis of lesion identification and risk stratification: carotid plaque morphology on ultrasound, for instance, stratifies ischemic stroke risk [17]. What has changed is the range of scales at which that geometry can be resolved, downward past the acoustic diffraction limit and outward from a single imaging plane to whole limbs.

Conventional B-mode ultrasound displays the cross section and wall structure of large vessels well, but the acoustic diffraction limit and poor sensitivity to slow flow leave microvascular networks invisible. Ultrafast Doppler recovers part of this by combining plane-wave imaging with spatiotemporal filtering, reaching frame rates high enough to detect slow microvascular flow [12]. For example, it can resolve the synovial microvascular network of the metacarpophalangeal joint [18]. However, resolution remains limited by acoustic diffraction, which is insufficient for capillary level imaging. Super-resolution ultrasound (SRUS) breaks that diffraction limit by localizing and tracking individual acoustic scatterers. In ultrasound localization microscopy (ULM), microbubbles are localized and tracked to reconstruct vascular networks and to measure vascular density, diameter, and flow velocity. Two representative patient studies mark how far this has come: transthoracic ULM of the human coronary microcirculation [19], and transcranial ULM that mapped tortuous cerebral arterial networks at approximately 25 μm and detected flow vortices inside deep small aneurysms [13]. Contrast-free SRUS instead uses endogenous scatterers, mainly red blood cells and their clusters, thereby removing the need for an exogenous agent and shortening acquisition, which in turn reduces sensitivity to motion. Renal vessels of approximately 28 μm have been visualized with acquisitions of 1–10 s [20], and human lymph-node microvasculature down to 40 μm within 2 s [21]. More recently, ultrasound phase microscopy reports a resolution finer than 5 μm within seconds [22]. Complementing these advances in resolution and imaging speed, recent developments have increasingly extended into three dimensions, carrying these gains across entire vascular networks. Recent advances in three-dimensional ultrasound imaging have been demonstrated through capacitive micromachined transducer arrays for ultrafast 3D Doppler [23], optically tracked free-hand scanning for intraoperative cerebral imaging [24], and multi-lens arrays for whole-organ ULM in large animals [25].

**Fig. 1:**
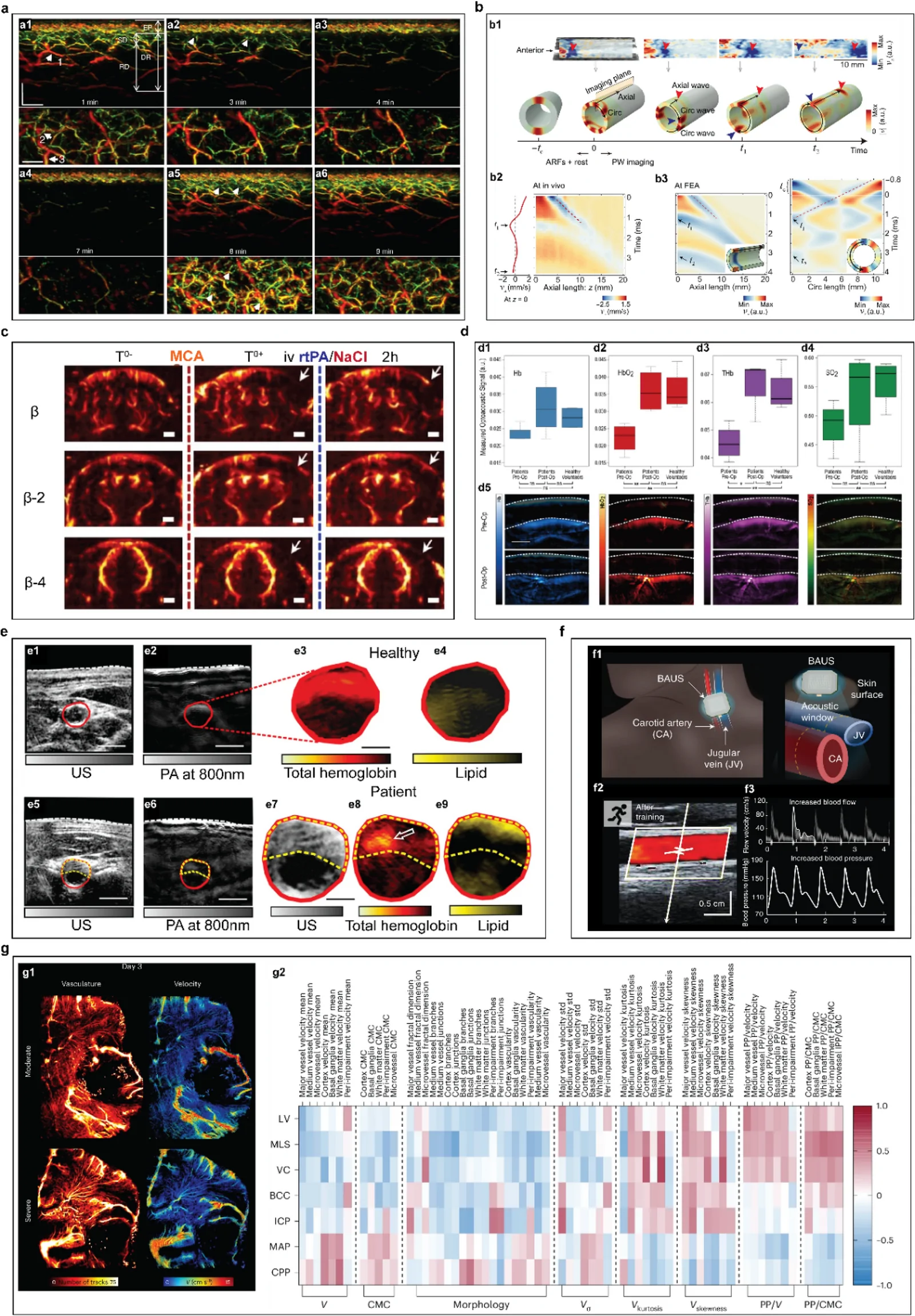
Representative advances in photoacoustic and ultrasound imaging for vascular phenotyping. **a**, Three-dimensional raster-scanning optoacoustic mesoscopy visualizes cutaneous microvascular dynamics during post-occlusive reactive hyperemia [27]. Each panel combines a cross-sectional view (top) with a dermal maximum-intensity projection (bottom): (a1) baseline; (a2–a4) cuff occlusion at 3, 4 and 7 min; and (a5, a6) reactive hyperemia at 8 and 9 min. *Arrowheads* in a5 indicate newly visualized vessels. **b**, Bidirectional guided-wave ultrasound characterizes arterial mechanical properties [32]. (b1) Time-resolved particle-velocity maps and schematics of axial and circumferential wave propagation. (b2) In vivo axial spatiotemporal velocity map at end-diastole. (b3) Finite-element simulations of axial and circumferential guided waves. These measurements support synchronous estimation of arterial anisotropic stiffness, wall stress and local blood pressure. **c**, Ultrafast Doppler imaging tracks cerebral perfusion after ischemic stroke in mice, showing hypoperfusion following thrombin-induced middle cerebral artery occlusion and reperfusion after recombinant tissue plasminogen activator treatment, with saline as the control [33]. **d**, Multispectral optoacoustic tomography assesses calf-muscle oxygenation in peripheral arterial disease before and after revascularization [34]. (d1–d4) Deoxygenated hemoglobin, oxygenated hemoglobin, total hemoglobin and oxygen saturation, respectively. (d5) Corresponding spatial maps before and after intervention, supporting detection of the treatment-associated increase in oxygenated hemoglobin. **e**, Co-registered ultrasound and multispectral photoacoustic tomography combine vascular anatomy with hemoglobin and lipid contrast [35]. (e1, e2) Ultrasound and 800-nm optoacoustic images from a healthy volunteer. (e3, e4) Total-hemoglobin and lipid distributions in the healthy artery. (e5, e6) Corresponding images from a patient with carotid atherosclerosis. (e7–e9) Magnified ultrasound, total-hemoglobin and lipid views of the plaque. **f**, Bioadhesive ultrasound enables sustained vascular monitoring and captures exercise-induced hemodynamic changes [36]. (f1) Device placement over the carotid artery and jugular vein. (f2) Carotid color-flow image after exercise. (f3) Increased carotid flow velocity and blood pressure after exercise. **g**, Ultrasound localization microscopy enables quantitative cerebrovascular phenotyping after traumatic brain injury [37]. (g1) Postoperative-day-3 vascular and velocity maps from patients with moderate and severe injuries. (g2) Correlations between ultrasound-localization-microscopy-derived vascular features and clinical and physiological variables, linking cerebral microvascular structure and flow to injury status. Panel (**a**) was adapted from He et al. [27] under the Creative Commons Attribution 4.0 International License. Panel (**b**) was adapted from Jiang et al. [32] under the Creative Commons Attribution 4.0 International License, © 2025 The Authors, some rights reserved; exclusive licensee American Association for the Advancement of Science. Panel (**c**) was adapted from Hingot et al. [33] under the Creative Commons Attribution 4.0 International License. Panel (**d**) was adapted with permission from Karlas et al. [34], © 2021 Annals of Translational Medicine. All rights reserved. Panel (**e**) was adapted with permission from Karlas et al. [35], © 2021 Elsevier GmbH. Panel (**f**) was adapted with permission from Wang et al. [36], © 2022 The Authors, some rights reserved; exclusive licensee American Association for the Advancement of Science. Panel (**g**) was adapted with permission from Li et al. [37], © 2026 The Author(s), under exclusive licence to Springer Nature Limited.

Photoacoustic imaging, in contrast, forms images from the optical absorption of endogenous hemoglobin. Its spatial scale extends from micron-scale resolution over a single capillary at one end, to sub-millimeter to millimeter resolution over the limbs, breast, and brain at the other, with field of view and penetration set by system configuration [15]. At the microscopic to mesoscopic scale, photoacoustic imaging of forearm dermal microvasculature distinguishes patients with carotid artery disease from healthy controls [26], and resolves endothelial dysfunction at the level of individual capillaries [27] (Fig. 1a), an early marker of vascular impairment. A fast all-optical three-dimensional photoacoustic scanning system captures corkscrew and tortuous vessels in the feet of patients with diabetes [28]. Imaging speed determines what else becomes visible: fast photoacoustic microscopy tracks vasoconstriction dynamics as it develops during acute hyperglycemia [29]. For cerebral vascular imaging, ultrafast functional photoacoustic microscopy achieved high-resolution imaging of cortical microvasculature, cerebral hemodynamics, and oxygenation [30]. Similarly, super-resolution methods based on red blood cell localization push the resolution further for microvascular imaging [31].

At the organ scale, photoacoustic computed tomography (PACT) trades resolution for depth. Breast imaging has been the main proving ground, where single-breath-hold PACT produced contrast comparable to Gd-enhanced MRI without exogenous agents [38, 39]. In the periphery, PACT delineated the vasculature around foot ulcers and supplied imaging evidence for wound blood supply and healing potential [40], while a semicircular-array system reconstructed the three-dimensional peripheral vascular network in peripheral artery disease (PAD) [41]. Recent PACT advances have improved effective aperture and view coverage to enhance image completeness and resolution, for example by extending the detection surface with a synthetic large aperture matrix [42] and by increasing angular coverage and suppressing limited-view artifacts through rotational scanning [43]. These high-quality structural images are also what feature extraction and machine-learning analysis operate on, which we return to below.

What none of these studies yet report is whether these added morphological details change a clinical management decision. One direct opportunity is at the foot: assessment of wound blood supply and healing potential [40] maps onto a choice clinicians make routinely, between conservative care, revascularization, and amputation. However, establishing that link will require studies designed around that endpoint rather than around image quality.

#### Blood Vessel Wall Mechanics: From Regional Stiffness to a Local Map

Arterial stiffness predicts cardiovascular events independently of conventional risk factors [44, 45], which is why wall mechanics matter clinically. Ultrasound has measured them in two ways. Wall tracking combines pulsatile diameter change with a pressure measurement to estimate local distensibility and compliance [10]. Regional pulse wave velocity, measured between two arterial sites, reports the average stiffness of everything in between [46]. Pulse wave imaging tracks the pulse wave along an arterial segment at high frame rate, yielding local pulse wave velocity and the corresponding elastic modulus along the vessel [47, 48]. Vector flow imaging reconstructs two- or three-dimensional velocity vectors and from them estimates wall shear stress, the mechanical load that flow imposes on the wall [49]. The two can be acquired together, giving stiffness and wall shear stress from one measurement [48]. Because an artery is a thin-walled waveguide loaded by blood pressure, its stiffness is neither isotropic nor pressure-independent, and recent work addresses both directly: bidirectional guided waves excite the anterior and posterior walls and return anisotropic stiffness, wall stress, and local blood pressure from a single acquisition [32] (Fig. 1b). Because these measurements are noninvasive and repeatable, they are also the ones now being moved onto wearable transducers [50], which are discussed below.

Photoacoustic imaging entered this area later and from a different direction, through elasticity and viscoelasticity characterization of tissue generally [51–53], and was only recently applied to vessels. A biomechanical-model-based method quantified microvascular elasticity during microvascular calcification [54] while photoacoustic elastography has been integrated with ultrasound and photoacoustic imaging for multiparametric and multimodal assessment [55]. Two newer approaches extend what is measurable: photoacoustic shear wave elastography reaches higher spatial resolution than conventional shear-wave elastography [56], and photoacoustic-strain imaging combines externally applied tissue deformation with ultrasound and photoacoustic imaging to quantify blood displacement and reperfusion dynamics [57]. The body of photoacoustic imaging work remains small, and the vascular applications remain preclinical.

Regional pulse wave velocity is already established as a clinical measure of arterial stiffness, which gives the local methods a clear question to answer: does a spatially resolved measurement of stiffness and wall stress reclassify intermediate-risk patients, or track response to antihypertensive therapy, better than a single regional number? That comparison has not yet been made.

#### Hemodynamics: Vessel-Resolved Flow and Perfusion

Ultrafast acquisition changed hemodynamic ultrasound from sequential line-by-line velocity measurements to a flow field across a region. Functional ultrasound maps perfusion: in a thromboembolic stroke model it tracked cerebral hypoperfusion and the reperfusion that followed recombinant tissue plasminogen activator [33] (Fig. 1c). Vector flow imaging, introduced above for wall shear stress, also reduces the angle dependence that limits conventional Doppler ultrasound, which is what allows complex flow near bifurcations, stenoses, and plaques to be resolved [48]. ULM contributes flow quantification vessel by vessel rather than in aggregate in human studies: transcranial ULM characterized flow dynamics in deep cerebral vessels in adults [13], and transthoracic ULM mapped myocardial microvasculature and flow direction [19]. Wearable transducers add the time axis, with synchronized patches used to detect combined coronary and carotid disease [58].

Photoacoustic imaging has also moved from static vessel display toward flow and volume dynamics. Photoacoustic vector tomography uses the intrinsic absorption heterogeneity of blood to recover flow vectors at approximately 5 mm depth [59]. A single-element ultrafast volumetric method follows superficial vessel hemodynamics longitudinally through an occlusion challenge [60]. Recently, a miniaturized omnidirectional broadband photoacoustic imaging system achieved continuous coverage from superficial to deep subcutaneous vessels and could capture changes in vessel diameter, hemoglobin concentration, and blood oxygenation under different physiological states, supporting continuous in vivo monitoring of microvascular dynamics [61].

The two divide the work cleanly: ultrasound holds the advantage in depth, velocity range, and continuous acquisition, while photoacoustic imaging reports microvascular perfusion and oxygenation that ultrasound cannot measure, without contrast agents. The reperfusion experiment points to where this matters clinically. Perfusion, not anatomy, is what thrombolysis and revascularization are meant to restore, and a bedside measurement of whether perfusion recovered would inform decisions now made from symptoms and delayed imaging. For cerebral reperfusion that measurement has been shown in a mouse model and not yet in patients.

#### Oxygenation: From Vessel Anatomy to Oxygen Delivery

Oxygenation is the phenotype ultrasound cannot reach. Because oxygenated and deoxygenated hemoglobin absorb light differently, multi-wavelength photoacoustic acquisition enables estimation of oxygen saturation. Co-registration with ultrasound places these photoacoustic measurements on the vascular anatomy from ultrasound imaging.

At the preclinical level, a photoacoustic microscopy system imaging the whole mouse brain at microvascular resolution and a 2 Hz volumetric rate captured oxygenation changes during hypoxia and spreading depolarization [30]. A head-mounted fiber endoscope extended the measurement to freely moving mice, which showed rising cerebral oxygenation while inhaling carbon dioxide [62]. An integrated two-photon and photoacoustic microscope resolved neuronal calcium activity and oxygen release from single red blood cells simultaneously [63].

In patients, a functional PACT system built on a hemispherical parallel transducer array imaged the human brain after craniectomy, with spatial correspondence to 7 T BOLD functional MRI over the same field of view, and it quantified oxygen saturation and blood volume directly [64]. By integrating multi-wavelength light sources, researchers achieved blood oxygenation assessment at the level of single vessels and carried out assessment during post-occlusive reactive hyperemia testing on the forearm and proximal nail fold [65]. In PAD, a major manifestation of CVD, a pilot photoacoustic study separated patients from healthy volunteers on calf-muscle oxygenation and detected a rise in oxygenated hemoglobin after revascularization [34] (Fig. 1d). Post-exercise calf-muscle oxygen saturation was then derived and validated in independent cohorts as a noninvasive biomarker of intermittent claudication [66], and calf-muscle hemoglobin content tracked disease severity [67].

In addition, the co-registered structural and functional imaging provided by combining super-resolution ultrasound with photoacoustic imaging is a notable trend that has so far been validated and demonstrated in mice. For example, a photoacoustic and ULM system quantified cerebral blood perfusion, oxygen saturation, vascular density, and flow velocity in murine ischemic stroke, showing stroke-induced ischemia, hypoxia, and reduced blood flow [68]. Another photoacoustic and ULM system demonstrated microvascular angiography of the mouse kidney with tissue oxygenation [69]. Together, these represent the most complete microvascular phenotypes that either modality has produced, and this combined approach has not yet been attempted in patients.

### Pathological Characterization

Plaque composition changes as the blood vessel wall remodels, and the two modalities read that change through different physics. Photoacoustic imaging separates tissue components by their optical absorption spectra. Ultrasound reports the acoustic consequences, echogenicity and texture, together with how the plaque interacts with flow [48, 49, 70].

Atherosclerosis is a lipid-driven disease of the artery wall, and plaque stability is closely associated with the size and distribution of lipid cores [3, 71]. Early work established that photoacoustic imaging can detect plaque lipid [72–75], and multispectral analysis separated lipid signals from the spectral background of heterogeneous plaque, which made lipid-rich regions localizable in human tissue [35, 76]. In multimodal approaches, ultrasound provides structural context that complements the compositional information provided by photoacoustic imaging, enabling more comprehensive characterization [35] (Fig. 1e). In the coronary arteries, intravascular photoacoustic and ultrasound imaging pairs depth-resolved lipid contrast with co-registered wall morphology [77]. Recent catheter development has targeted lipid sensitivity, localization, and acquisition speed, through dual-frequency detection [78], graded-index-fiber optical delivery [79], and miniaturized high-speed rotational systems [80].

Beyond lipid accumulation, intraplaque neovascularization (IPN) and intraplaque hemorrhage (IPH) represent additional and complementary indicators of plaque vulnerability. Ultrasound detects IPN by visualizing blood flow within the plaque [81]. Contrast-Enhanced Ultrasound (CEUS) and superb microvascular imaging provide semi-quantitative grades that agree well with histopathological neovascularization [82], while clinical and meta-analytic evidence supports their diagnostic value and the association of IPN with coronary artery disease [81, 83, 84]. ULM extends conventional CEUS from aggregate enhancement assessment to resolving plaque neovasculature and quantifying its flow [85, 86]. On the photoacoustic side, a 16-patient study showed IPH-related photoacoustic contrast in vivo [87]. Signal-processing approaches have further improved the separation of IPH-related signals from luminal blood [88], and multispectral photoacoustic imaging distinguished lipid from hemoglobin in human carotid plaque by their spectral signatures [35, 89].

The fibrous cap and inflammatory activity are harder targets. Multispectral PA analysis extracts collagen-related components from plaques [76], and combined PA and US imaging distinguished vulnerable regions in excised human carotid plaques [90], but direct in vivo measurement of cap thickness is still obstructed by luminal blood and surrounding tissue. Inflammation has little endogenous photoacoustic specificity, which is why the work here is probe-based: agents targeting macrophage and angiogenesis pathways including CD36 and αvβ3 integrin [91, 92], and activatable, ratiometric, and dual-enzyme-responsive designs that allow inflammatory activity to be quantified [93–95].

### From Measurement to Monitoring

Everything above is a measurement made once by an operator. Continuous measurement changes what can be asked. A single clinic blood pressure or a single duplex study describes one moment, while drug titration, detection of early graft or stent failure, and decisions about discharge all depend on trends. Two developments determine whether these photoacoustic and ultrasound-based phenotypes can instead be acquired repeatedly and read reproducibly. Wearable devices move acquisition from the examination room to continuous monitoring. Feature extraction converts image patterns into numbers that transfer between operators and centers. Neither adds a new contrast mechanism; both decide whether the contrasts already described can support clinical decisions.

#### Wearable Systems: Continuous, Operator-Independent Acquisition

Conventional photoacoustic and ultrasound systems are bulky and operator dependent. Wearable versions address these limitations, and in doing so reach transient and evolving vascular changes that a scheduled examination cannot catch [96, 97]. Three problems have organized the work: holding signal quality steady over hours and during body motion, reaching deeper tissue and more phenotypes, and packaging both into miniaturized, flexible, wireless hardware [36, 96, 98, 99].

Arterial waveforms are an accessible entry point and are informative about cardiovascular physiology [11]. Conventional measurements are sensitive to probe position and angle, relative displacement, and variable acoustic coupling. A stretchable skin-conformal ultrasound patch enables continuous measurements of central arterial blood-pressure waveforms [100], and Doppler patches use different angle-estimation strategies to reduce the dependence on insonation angle and on repeated operator alignment [101, 102]. A carotid Doppler patch tracked stroke-volume changes through velocity–time integrals in healthy volunteers, providing proof-of-concept evidence for hands-free cardiac-output trending [103]. Prolonged monitoring then becomes a question of the skin–device interface, where bioadhesive ultrasound holds coupling with a hydrogel–elastomer couplant for up to 48 h of continuous vascular and cardiac imaging [36] (Fig. 1f). Building on improved coupling, a skin-conformal phased array focuses and steers actively, monitoring hemodynamic signals up to 14 cm below the skin [104]. A stretchable orthogonal phased-array patch with curvature-aware beamforming carries wearable ultrasound toward quantitative echocardiography, with chamber geometry, myocardial motion, and ventricular performance now measurable continuously [96, 97]. Three-dimensional and shear-wave wearable ultrasound widen the accessible phenotypes further toward tissue mechanics, although their cardiovascular application is still prospective [105, 106].

Many recent works in this area operate continuously without supervision. A fully integrated system combines miniaturized electronics, wireless communication, and machine-learning target tracking to hold deep-tissue signals during body motion [99], and skin-adaptive focused flexible transducers accommodate skin curvature and target depth while tracking blood-pressure waveforms, exercise heart rate, and arterial stiffness [98]. Another clinical demonstration is that a wearable ultrasound blood-pressure sensor has been validated across daily activity at home, outpatient clinics, cardiac catheterization laboratories, and intensive care units [107]. Synchronized ultrasound patches also allow cardiac and vascular dynamics to be jointly interpreted incoronary–carotid comorbidity [58].

Wearable photoacoustic imaging adds what wearable ultrasound cannot achieve: hemoglobin concentration, oxygenation, and high microvascular contrast [108, 109]. A photoacoustic patch with an integrated miniaturized light source maps hemoglobin three-dimensionally in deep tissue [108], while other flexible photoacoustic patches extended wearable measurement to oxygenation, vascular function, and microvascular structure [109, 110]. A wrist-worn system brings high-resolution microvascular imaging into ambulatory use [110]. Light-source miniaturization and system integration remain the main barriers to prolonged and fully mobile PA monitoring. Most of these systems are proof-of-concept, and none has been tested inside a clinical care pathway.

#### Feature Extraction: From Images to Reportable Metrics

Feature extraction is what converts an image into a number that another operator, at another center, can reproduce. Photoacoustic and ultrasound interpretation is still largely reader-dependent and sensitive to image quality, and much of what the images contain is not perceptible to a reader at all. Extracting it moves vascular assessment from description toward quantitative measurement.

Predefined features encode prior knowledge about what matters. From ultrasound microvascular imaging, fractal dimension, branching characteristics, and spatial vascularity patterns quantify network alterations [111]. ULM parameters capture microvascular dynamics tied to neurovascular activity and to cerebrovascular remodeling after traumatic brain injury [37, 112] (Fig. 1g). In preclinical studies, ULM features characterized pericyte-dependent microvascular regulation [113].

Photoacoustic feature extraction spans different scales. At the microscopic scale, raster-scan photoacoustic mesoscopy captures depth-resolved vascular networks across the skin, and machine-learning pipelines applied to them extracted vascular density, depth, branching, network organization, and epidermal features that stage diabetic microangiopathy quantitatively [114, 115]. Adding a post-occlusive reactive hyperemia challenge moves the features from static morphology to reactivity, which separated patients with carotid artery disease from healthy controls [26]. At the macroscopic level, panoramic photoacoustic computed tomography enabled whole-breast vascular phenotyping and applied learning-based analysis to vascular patterns for distinguishing benign from malignant lesions [116].

Two extensions matter more for cardiovascular use than added spatial coverage. Longitudinal acquisition tracks change: signal intensity, texture, and vascular morphology followed over time revealed outcome-associated trajectories in chronic foot ulcers [117]. Multi-wavelength acquisition adds function: multispectral calf-muscle biomarkers, oxyhemoglobin, deoxyhemoglobin, and oxygen saturation, separated patients with intermittent claudication from healthy controls [66]. These are features closely tied to an outcome and to a physiological quantity rather than to image appearance.

Additionally, combining the modalities produces features neither yields alone. This has been shown most extensively outside the cardiovascular setting, in breast imaging, where photoacoustic vascular features added to ultrasound morphology improved lesion assessment [118] and volume-wide co-registered acquisition supported integrated structural and functional interpretation [119]. The methodology transfers even though the indication does not.

Three limitations stand between these features and their use as biomarkers. Features are largely application-specific, and validation across diverse patient populations is limited. The biology underlying most of them is not established. The last matters most for cardiovascular adoption: a feature that predicts an outcome without a mechanism is hard to act on when the action is a change in therapy. What would settle the first two is a multicenter study in which the same feature is extracted from the same patient on different systems by different operators, and the values agree. No photoacoustic or super-resolution ultrasound feature has been through that.

## Conclusions

This review highlights recent advances in ultrasound and photoacoustic imaging for CVD. Ultrafast, three-dimensional, and super-resolution ultrasound now resolve vascular morphology, microcirculation, wall biomechanics, and hemodynamics in patients, and photoacoustic imaging adds oxygenation, lipid deposition, intraplaque hemorrhage, inflammation, and other wall components through optical absorption contrast. Separately, wearable systems and feature extraction are turning single operator-dependent examinations into continuous, reproducible quantities. The first change widens what can be measured; the second determines whether those measurements can be obtained reproducibly.

Adoption will turn partly on regulatory fit, which differs between the two modalities. For microbubble-based ultrasound, approval of a contrast agent for an established indication does not extend to a new application, and evidence specific to the intended use will be required. Photoacoustic imaging, which mostly relies on endogenous contrast, avoids that problem but has no established clinical indication to build from, so each intended use will need its own validation against defined performance standards.

Both modalities still face several common challenges: quantitative consistency at depth, robustness to motion, hardware miniaturization, and standardization across centers. None of these is a discovery problem. The harder requirement is that emerging imaging biomarkers be tied to endpoints clinicians already act on, and the field has so far been better at improving images than at designing studies around decisions. The photoacoustic and ultrasound measurements need to be chosen because they answer a question a clinician faces, which would make photoacoustic and advanced ultrasound imaging part of how cardiovascular disease is managed rather than how it is described.

## Key References

- He H, Karlas A, Fasoula NA, Fischer C, Darsow U, Kallmayer M, et al. Single-capillary endothelial dysfunction resolved by optoacoustic mesoscopy. Light Sci Appl. 2026;15:37.
  - ○ This study introduces fast raster-scan optoacoustic mesoscopy for non-invasive assessment of cutaneous microvascular endothelial function at single-capillary resolution, revealing layer-specific dysfunction in smokers and patients with atherosclerotic cardiovascular disease before substantial structural vascular changes are detectable.
- Wang C, Chen X, Wang L, Makihata M, Liu H-C, Zhou T, et al. Bioadhesive ultrasound for long-term continuous imaging of diverse organs. Science. 2022;377:517–23.
  - ○ This study develops a bioadhesive ultrasound device capable of providing 48 hours of continuous, high-resolution imaging of blood vessels and multiple internal organs, establishing a foundational wearable ultrasound platform for long-term physiological and disease monitoring.
- Li M, Wang W, Su Z, Wu J, Yang Y, Wang L, et al. Clinically translatable ultrasound localization microscopy reveals cerebrovascular remodelling and prognosis in patients with traumatic brain injury. Nat Biomed Eng. 2026;1–17.
  - ○ This prospective clinical study demonstrates bedside ultrasound localization microscopy in patients with traumatic brain injury, enabling the quantification of severity-dependent cerebrovascular remodelling and identifying microvascular metrics associated with intracranial pressure and 6-month clinical outcomes.

## Data Availability

No datasets were generated or analysed during the current study.

## Declarations

### Use of Artificial Intelligence

Artificial intelligence (AI) tools were used solely for language and grammar editing. AI was not used to generate scientific content, analyze or interpret data, create figures or tables, or draw conclusions. All content was reviewed and approved by the authors, who take full responsibility for the final manuscript.
